# Determinants at the N- and C-termini of Gα_12_ required for activation of Rho-mediated signaling

**DOI:** 10.1186/1750-2187-8-3

**Published:** 2013-03-25

**Authors:** Benjamin J Ritchie, William C Smolski, Ellyn R Montgomery, Elizabeth S Fisher, Tina Y Choi, Calla M Olson, Lori A Foster, Thomas E Meigs

**Affiliations:** 1 Department of Biology, University of North Carolina at Asheville, One University Heights, Asheville, NC 28804, USA

**Keywords:** Gα_12_, Gα_13_, Heterotrimeric G protein, RhoGEF, Rho, LARG, Serum response element

## Abstract

**Background:**

Heterotrimeric guanine nucleotide binding proteins of the G12/13 subfamily, which includes the α-subunits Gα_12_ and Gα_13_, stimulate the monomeric G protein RhoA through interaction with a distinct subset of Rho-specific guanine nucleotide exchange factors (RhoGEFs). The structural features that mediate interaction between Gα_13_ and RhoGEFs have been examined in crystallographic studies of the purified complex, whereas a Gα_12_:RhoGEF complex has not been reported. Several signaling responses and effector interactions appear unique to Gα_12_ or Gα_13_, despite their similarity in amino acid sequence.

**Methods:**

To comprehensively examine Gα_12_ for regions involved in RhoGEF interaction, we screened a panel of Gα_12_ cassette substitution mutants for binding to leukemia-associated RhoGEF (LARG) and for activation of serum response element mediated transcription.

**Results:**

We identified several cassette substitutions that disrupt Gα_12_ binding to LARG and the related p115RhoGEF. These Gα_12_ mutants also were impaired in activating serum response element mediated signaling, a Rho-dependent response. Most of these mutants matched corresponding regions of Gα_13_ reported to contact p115RhoGEF, but unexpectedly, several RhoGEF-uncoupling mutations were found within the N- and C-terminal regions of Gα_12_. Trypsin protection assays revealed several mutants in these regions as retaining conformational activation. In addition, charge substitutions near the Gα_12_ N-terminus selectively disrupted binding to LARG but not p115RhoGEF.

**Conclusions:**

Several structural aspects of the Gα_12_:RhoGEF interface differ from the reported Gα_13_:RhoGEF complex, particularly determinants within the C-terminal α_5_ helix and structurally uncharacterized N-terminus of Gα_12_. Furthermore, key residues at the Gα_12_ N-terminus may confer selectivity for LARG as a downstream effector.

## Background

The G12/13 subfamily of heterotrimeric guanine nucleotide binding proteins (G proteins) is comprised of two α-subunits in mammals, Gα_12_ and Gα_13_, that have been implicated in a variety of physiological and pathological cellular responses that include proliferation, cytoskeletal rearrangements, migration, and metastatic invasion [1,2]. A diverse set of putative effector proteins have been identified as direct interactors with one or both G12/13 subfamily members; however, the roles of individual Gα-effector interactions in specific cellular responses remain largely undefined [3]. The most extensively characterized G12/13 target proteins are a subset of Rho-specific guanine nucleotide exchange factors (RhoGEFs) that activate the monomeric G protein Rho via tandem Dbl-homology/pleckstrin-homology domains [4]. The Rho monomeric GTPases are known primarily for their role in regulating actin cytoskeletal dynamics, but these proteins also mediate cell polarity, microtubule dynamics, membrane transport pathways, transcription factor activity, cell growth, and tumorigenesis [5]. The G12/13-RhoGEF-Rho axis mediates critical signaling and developmental pathways in model organisms that include *Drosophila melanogaster*[6], *Caenorhabditis elegans*[7], and zebrafish [8]. In addition, direct interaction with RhoGEFs is required for mutationally activated Gα_12_ to trigger increased invasiveness of breast cancer cells [9].

Activated G12/13 α-subunits trigger Rho activation via binding and stimulation of three distinct RhoGEFs: p115RhoGEF, LARG and PDZ-RhoGEF [10-13]. This interaction is mediated primarily by a domain, located near the N-terminus of each RhoGEF, that is closely related to the regulator of G protein signaling (RGS) domain that defines the growing family of RGS proteins [14,15]. Although p115RhoGEF, LARG and PDZ-RhoGEF are highly similar in this “RGS homology“ (RH) domain [16], these proteins appear to be activated by different mechanisms and play non-redundant roles in G12/13 subfamily-mediated signaling. Purified p115RhoGEF binds Gα_12_ and Gα_13_ and accelerates GTPase activity for both proteins, but only Gα_13_ can stimulate p115RhoGEF to activate RhoA *in vitro*[10,17]. Interaction of Gα_12_ or Gα_13_ with purified LARG can trigger its activation of RhoA; however, stimulation by Gα_12_ requires prior phosphorylation of LARG by the nonreceptor tyrosine kinase Tec [13]. Furthermore, studies utilizing small interfering RNA to hinder expression of specific RhoGEFs show that LARG is a specific downstream effector of thrombin receptor-mediated signaling, whereas signaling through the lysophosphatidic acid (LPA) receptor is attenuated by blocking PDZ-RhoGEF expression [18]. These results are compelling in light of a separate report that the thrombin receptor shows preferential coupling to Gα_12_, whereas the LPA receptor preferentially utilizes Gα_13_ as a conduit to downstream signaling [19]. Although it is possible that Gα_12_ stimulates a post-translationally modified form of p115RhoGEF or PDZ-RhoGEF in cells, the evidence to date suggests LARG as the most likely RhoGEF serving as a physiological effector for Gα_12_. Gains in our understanding of the specificity of RhoGEF engagement within the G12/13 subfamily should provide insights into the non-overlapping functions of Gα_12_ and Gα_13_ in signal transduction.

Crystallographic studies have revealed important structural aspects of the interaction between Gα_13_ and the RH domain of p115RhoGEF, including numerous residues in both proteins that provide contact points [20,21]. Initially, purification of Gα_13_ for crystallography required that it be engineered as a chimera in which amino acid sequence within several regions, including the N- and C-termini, was replaced by corresponding sequence from the Gi subfamily protein Gαi_1_[20]. The structure of the Gα_13_:p115RhoGEF-RH complex was later refined in crystallographic studies that utilized a Gα_13_ chimera harboring Gαi_1_ sequence only at the N-terminus. Because the Gα N-terminus was unstructured in this crystallized complex, any role of this region in RhoGEF interaction remains to be determined. Although the region of Gα_13_ downstream of the Switch III region harbors several residues critical for RhoGEF engagement, notably Glu^273^, Thr^274^, Asn^278^, and Arg^279^ within the α_3_ helix and α_3_-β_5_ loop, other regions closer to the Gα_13_ C-terminus do not emerge in the crystal structure as providing key RhoGEF contact points [21].

In contrast to Gα_13_, a structure of Gα_12_ in complex with a RhoGEF target has not been reported, although a chimeric Gα_12_ harboring the N-terminus of Gαi_1_ has been crystallized [22]. To examine the full sequence of Gα_12_ for structural features mediating its interaction with RhoGEFs, we engineered a series of cassette substitutions within constitutively activated Gα_12_ and examined these variants for *in vitro* binding to the RH domains of LARG and p115RhoGEF, as well as ability to drive the Rho-dependent process of serum response element (SRE) mediated transcription in cells [23]. Our results reveal unexpected regions of Gα_12_ as harboring determinants of its functional interaction with RhoGEFs, and also identify key charged amino acids near the Gα_12_ N-terminus that may confer selective binding to LARG.

## Results

### Myc-tagged Gα_12_ retains RhoGEF binding, Rho-mediated signaling, and conformational activation

**Figure 1:**
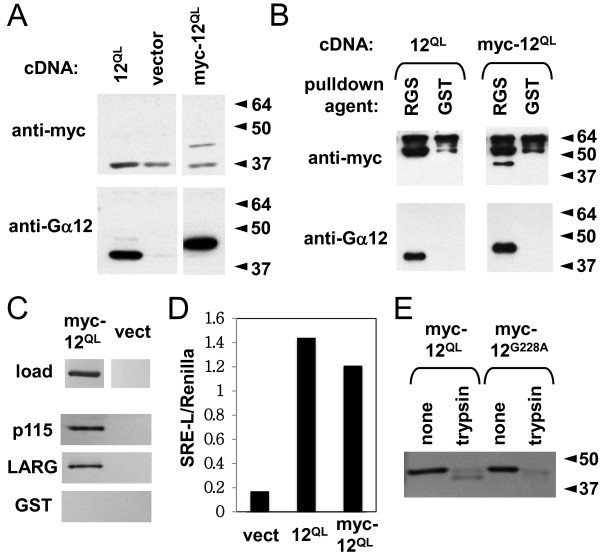
**Effector binding and conformational activation of myc-tagged, constitutively activated Gα**_**12**_**.** Molecular weight markers (in kDa) are indicated at right of panels where applicable. All results shown are representative of two or more independent experiments. (**A**) Expression and solubilization of Gα_12_^QL^ (*12*^*QL*^) and myc-tagged Gα_12_^QL^ (*myc-12*^*QL*^) transiently expressed in HEK293 cells. Cells transfected with the vector pcDNA3.1 are included as a negative control (*vector*). Detergent-soluble extracts were prepared by high-speed centrifugation and subjected to SDS-PAGE and immunoblotting, using either anti-myc (Zymed) or anti-Gα_12_ (Santa Cruz Biotechnology) antibodies as described in Methods. (**B**) *In vitro* binding of myc-tagged and untagged Gα_12_^QL^ by p115RhoGEF. HEK293 cells extracts containing myc-Gα_12_^QL^ were subjected to protein interaction assays (see Methods) using an immobilized GST fusion of the RH domain of p115RhoGEF (*RGS*) or GST alone (*GST*). Samples were washed, separated by SDS-PAGE, and analyzed by immunoblotting using antibodies described above. (**C**) Specificity of myc-Gα_12_^QL^ detection in interaction assays. HEK293 cells transfected with either myc-Gα_12_^QL^ (*myc-12*^*QL*^) or the empty pcDNA3.1 plasmid (*vect*) were lysed and assayed for binding to GST fusions of the RH domain of p115RhoGEF (*p115*) or LARG, or GST alone (*GST*). Immunoblot analysis was performed using anti-Gα_12_ antibody as described above. (**D)** Serum response element (SRE) luciferase activation by myc-Gα_12_^QL^. HEK293 cells grown in 12-well plates were co-transfected with the plasmids SRE-L (0.2 μg) and pRL-TK (0.02 μg), plus 0.1 μg of the plasmid indicated on the X-axis. Y-axis values show firefly luciferase signal normalized for *Renilla* luciferase signal within each sample. (**E**) Trypsin protection assays of myc-tagged Gα_12_. Lysates from HEK293 cells transfected with myc-Gα_12_^QL^ (*myc-12*^*QL*^) or the constitutively GDP-bound Gly^228^Ala mutant of wildtype Gα_12_ (*myc-12*^*G228A*^) were subjected to trypsin digests as described in Methods. Immunoblot analysis was performed using J169 antibody [25] at 1:700 dilution.

To identify mutants of Gα_12_ impaired in RhoGEF binding, we first sought to establish an *in vitro* system in which Gα_12_ mutants could be expressed ectopically in cultured cells, rendered soluble in a detergent extract, and detected without interference from endogenous Gα_12_. We engineered the constitutively active Gln^229^Leu variant of Gα_12_ (Gα_12_^QL^) to harbor a myc epitope tag, flanked by linkers of the sequence SGGGGS and positioned between residues Pro^139^ and Val^140^. This insertion site was chosen due to its approximate alignment with the position of green fluorescent protein in Gαq in a prior study [24]. We expressed myc-tagged and untagged Gα_12_^QL^ in HEK293 cells, prepared detergent-soluble extracts, and analyzed these by immunoblotting. As shown in Figure 1A, myc-tagged Gα_12_^QL^ was detected by both anti-myc and anti-Gα_12_ antibodies, with the latter generating a much stronger signal while avoiding an off-target 37 kDa band detected in all samples by the anti-myc antibody. Also, the myc-tagged protein (~45 kDa) was readily discernible from endogenous Gα_12_ and untagged Gα_12_^QL^ (~43 kDa). Next, we subjected myc-Gα_12_^QL^ to pulldown experiments using an immobilized GST fusion of the p115RhoGEF RH domain, as described in Methods. Myc-tagged and untagged Gα_12_^QL^ bound to p115-RH with similar affinity (Figure 1B), and comparison with mock-transfected cells indicated the ~45 kDa band detected by anti-Gα_12_ was dependent on transfection with the myc-Gα_12_^QL^ plasmid. Furthermore, LARG-RH and p115-RH showed similar ability to co-precipitate myc-tagged Gα_12_^QL^ (Figure 1C). To ascertain that myc-Gα_12_ is functional as a mediator of cellular signal transduction through Rho, we measured transcriptional activation of a luciferase reporter gene positioned downstream of the serum response element (SRE), a component of the c-fos promoter that provides a readout of Gα_12_-mediated Rho activation [23]. Myc-tagged and untagged Gα_12_^QL^ exhibited similar ability to stimulate this response in HEK293 cells co-transfected with SRE-luciferase (Figure 1D). Furthermore, trypsin digestion of HEK293 cell lysates harboring myc-Gα_12_^QL^ yielded a protected fragment of ~40 kDa, comparable to results observed previously with GTPγS-loaded, purified Gα_12_[25]. An inactive, constitutively GDP-bound (Gly^228^Ala) variant of myc-tagged Gα_12_ did not yield this ~40 kDa fragment when digested with trypsin (Figure 1E). Taken together, these results suggest myc-Gα_12_^QL^ undergoes conformational activation and retains normal signaling through the RhoGEF:Rho pathway. Because of the superior sensitivity of anti-Gα_12_ antibody in detecting myc-Gα_12_^QL^, and the easily discernible gel shift of Gα_12_ caused by the myc tag and linkers (see Figures 1A and B), we chose to utilize anti-Gα_12_ to detect myc-Gα_12_^QL^ in subsequent protein binding experiments.

### Mutations that uncouple Gα_12_ from RhoGEF binding and Rho-mediated signaling

**Figure 2:**
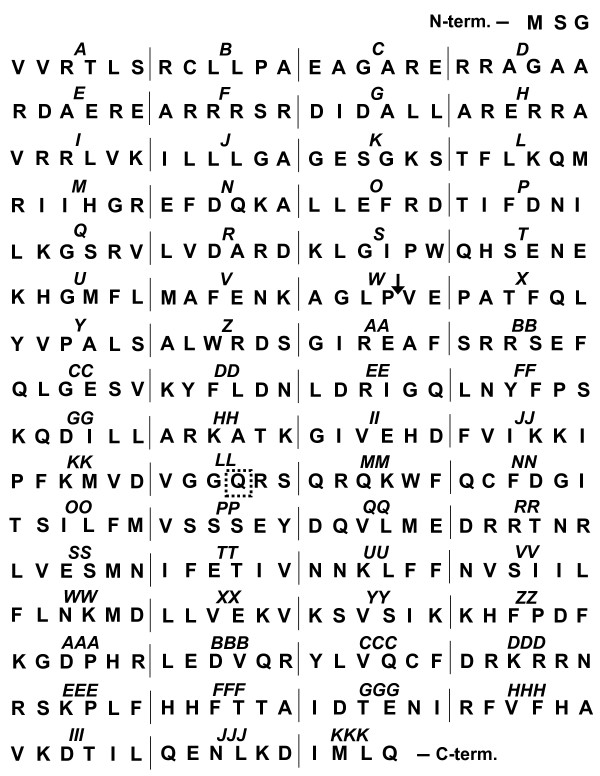
**Residues replaced in Gα**_**12 **_**cassette mutants.** For each mutant, designated in italics (*A*-*Z*, *AA*-*ZZ*, *AAA*-*KKK*), the native amino acid sextet replaced by the sequence Asn-Ala-Ala-Ile-Arg-Ser is shown. An arrow between Pro^139^ and Val^140^ indicates the site of myc tag insertion. Mutant *W* was not produced. The dashed box indicates the native Gln^229^ mutated to Leu to render Gα_12_ constitutively active. The native residues replaced in mutant *KKK* are Lys-Asp-Ile-Met-Leu-Gln and thus partially overlap with mutant *JJJ*. All cassette mutants contain the activating Q^229^L mutation, except mutant *LL* due to its cassette substitution.

**Figure 3:**
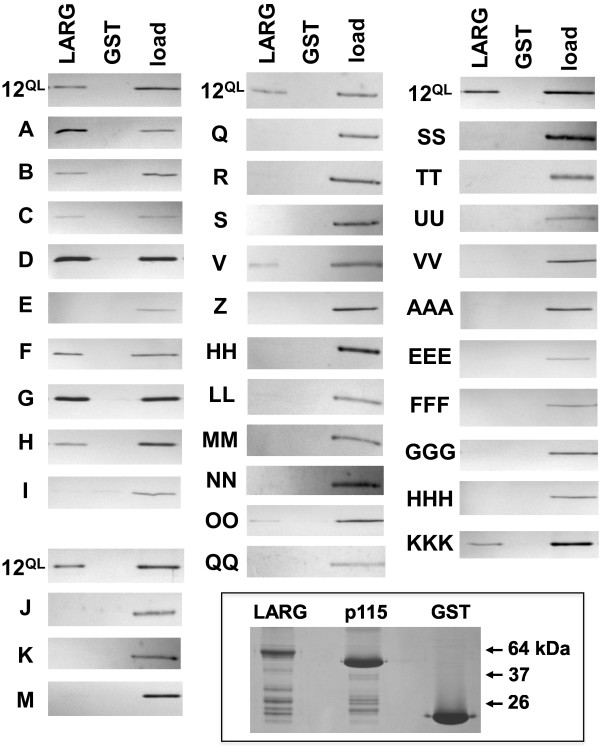
**In vitro interaction of Gα**_**12 **_**mutants with LARG.** Immunoblot results for all LARG binding-impaired Gα_12_ cassette mutants and selected other mutants are shown. HEK293 cells were transfected with the indicated plasmids (7.0 μg per 10-cm plate) and lysates were prepared for co-precipitation assays as described in Methods. Prior to this step, 5% of each lysate was set aside as starting material (*load*). Pulldown experiments were performed on 7–9 mutants per experiment, plus myc-Gα_12_^QL^ as a positive control, using equal amounts of GST-LARG-RH (*LARG*) immobilized on glutathione-sepharose. Immobilized GST was utilized in parallel as a negative control. For all experimental samples, 20% of the volume was analyzed by SDS-PAGE and Coomassie blue staining to verify equal amounts of GST-LARG-RH and GST proteins in the precipitates (data not shown). Immunoblots displayed in this figure are representative of at least three trials per cassette mutant, except for mutants *A*-*D*, *F*-*H*, *V*, and *KKK* that showed minimal impairment in LARG binding after two trials. (**Inset)** Coomassie blue analysis of GST-fusion constructs expressed in bacteria and immobilized on glutathione-sepharose: GST-LARG-RH (*LARG*), GST-p115-RH (*p115*), and GST alone. Molecular weight standards (in kDa) are indicated at right.

To scan Gα_12_ for regions participating in its interaction with RhoGEFs, we utilized a comprehensive panel of mutants in which sextets of consecutive amino acids in myc-Gα_12_^QL^ are replaced by the sextet Asn-Ala-Ala-Ile-Arg-Ser (Figure 2 shows the native amino acid sextet and alphabetical designation for each mutant). This strategy of “NAAIRS” cassette substitutions was chosen due to prediction of this motif being tolerated in the three-dimensional structure of proteins [26], prior use of this approach in mapping functional regions of both retinoblastoma and the telomerase catalytic subunit [27,28], and our previous success employing this strategy to identify Gα_12_ determinants of binding to the scaffolding subunit of protein phosphatase-2A and the cytoplasmic tail of polycystin-1 [29,30]. Variants of Gα_12_ were expressed in HEK293 cells and tested for interaction with immobilized LARG-RH, as described in Methods. As shown in Figure 3, myc-Gα_12_^QL^ was co-precipitated by a GST fusion of LARG-RH but not by GST alone. Many of these cassette mutants yielded a moderate-to-robust signal in the LARG-precipitated fraction; however, a subset displayed a weak or absent signal (Figure 3). To assess impairment of LARG binding for each myc-Gα_12_^QL^ variant, we quantified the band intensity for each precipitated sample (*pulldown*), and divided this by the band intensity in the starting cellular extract (*load*). These calculations generated a “pulldown:load ratio” for each mutant, and also for the positive control myc-Gα_12_^QL^ that was tested in each experiment. Nearly all cassette mutants were solubilized by our detergent conditions and detected by immunoblotting; exceptions were mutant *W*, which we did not engineer due to overlap with the insertion site of the myc tag (see Figure 2), and mutant *CC* due to low expression levels that produced inconclusive results (data not shown). As shown in Table 1, the majority of cassette mutants exhibited pulldown:load ratios greater than 40% of the ratio determined for myc-Gα_12_^QL^. However, a number of mutants exhibited lower pulldown:load ratios (<20% of positive control) with a subset generating a ratio less than 10% of the positive control. For all samples, precipitation by immobilized GST yielded no Gα_12_ signal (Figure 3), indicating these mutants were not merely binding the GST-glutathione-sepharose complex nor forming insoluble aggregates under these *in vitro* conditions. Also, we examined the full panel of Gα_12_ cassette mutants for interaction with a GST fusion of the N-terminal 252 amino acids of p115RhoGEF (p115-RH). None of the LARG binding-impaired mutants (those with pulldown:load ratio <20% of positive control; see Table 1) yielded a signal intensity in the p115-RH precipitate that exceeded 50% of intensity for the positive control myc-Gα_12_^QL^ (data not shown).

**Table 1:** **Gα**_**12 **_**cassette mutants impaired in binding LARG-RH**

70-100%	A-D F-H L N O T-V X BB DD-FF II KK XX YY BBB DDD III-KKK
40-70%	H P GG JJ WW ZZ CCC
20-40%	Y AA PP
10-20%	I OO QQ-SS UU VV FFF
0-10%	E J K M Q R S Z HH LL-NN TT AAA EEE GGG HHH
N/D	W CC

Cassette substitution mutants of myc-Gα_12_^QL^ (see Figure 2 for alphabetical designations) were expressed in HEK293 cells and subjected to protein interaction assays using a GST-fusion of the RH domain of LARG as described in Methods, and for each mutant a pulldown:load ratio was determined and calculated as a percent (left column) of the same ratio for unmodified myc-Gα_12_^QL^ assayed in parallel. Each Gα_12_ mutant was analyzed in three independent experiments, except for mutants that appeared in the 70-100% category in two independent experiments.

**Figure 4:**
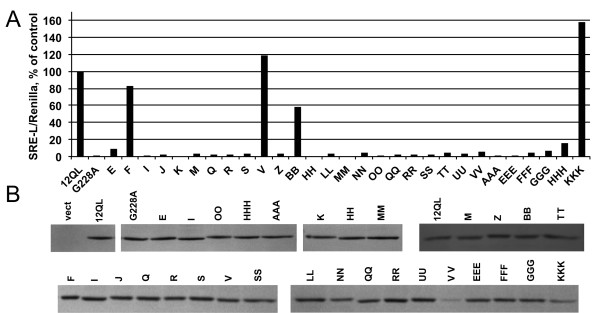
**Activation of serum response element mediated transcription by Gα12 mutants. **(**A**) Luciferase reporter assay results of selected cassette mutants. HEK293 cells grown in 12-well plates were co-transfected with the plasmids SRE-L (0.2 μg) and pRL-TK (0.02 μg), plus 1.0 μg of the plasmid encoding each cassette mutant indicated on the X-axis. Firefly luciferase values were normalized for *Renilla* luciferase values within each sample, and values are presented as a percent of the value calculated for myc-Gα_12_^QL^ (Y-axis) within the same experiment. Mutationally active (*12QL*) and inactive (*G228A*) samples were analyzed in parallel. Results shown are a representative of two experiments performed per Gα_12_ variant. (**B**) Expression level of Gα_12_ mutants. A sample of each lysate was set aside prior to luminometry and analyzed by SDS-PAGE and immunoblotting using anti-Gα_12_ antibody (Santa Cruz Biotechnology). For all samples, densitometric intensity was determined as described in Methods, then divided by positive control myc-Gα_12_^QL^ levels within the same experiment, and SRE-L/*Renilla* values were adjusted to reflect this normalization for protein levels.

The Gα_12_ cassette mutant designated *OO* was among those impaired in LARG binding, consistent with our previous work demonstrating its uncoupling from Rho-mediated signaling [31], and several other cassette substitutions within the Switch regions disrupted binding to LARG (mutants *HH*, *LL*, *MM*, *NN*, *QQ*, and *RR*; see Figure 2). However, impaired LARG binding also was caused by substitutions in other regions of Gα_12_ (Table 1). Prior crystallographic studies identified several residues in Gα_13_ that serve as contact points with p115-RH [20,21]. Table 2 lists Gα_13_ residues identified as contact points with p115-RH in these earlier studies, and indicates the corresponding Gα_12_ cassette mutant for each Gα_13_ residue. From our *in vitro* binding results (Table 1), it is apparent that most Gα_12_ mutants corresponding to RhoGEF-contacting Gα_13_ residues displayed partial or severe impairment of LARG binding, mutants *V, BB* and *DDD* being exceptions. However, several RhoGEF-uncoupling substitutions in Gα_12_ (cassette mutants *E, I, J, K, M*, *Z, NN, OO, VV, AAA, EEE, FFF, GGG* and *HHH*) replaced amino acids that do not correspond to Gα_13_ contacts with p115-RH. Gα_12_ mutants *J* and *K* replaced sections of the P-loop, a motif critical in guanine nucleotide binding, and thus would be predicted as impaired in signaling. However, our finding of RhoGEF-uncoupling mutations at the N- and C-termini of Gα_12_ was unexpected, because these regions either lacked corresponding contact points in the Gα_13_:p115-RH complex or were disordered in the G12/13 crystal structures (i.e. the N-terminus). To determine whether these N- and C-terminal mutations in Gα_12_ are impaired in Rho-mediated signaling, we expressed these variants in HEK293 cells and measured stimulation of SRE-luciferase transcription. All N- and C-terminal mutants impaired in RhoGEF binding were poor activators of this reporter gene (Figure 4A). Several cassette mutants in the N- and C-terminal regions of Gα_12_ that displayed normal binding to LARG (mutants *F, V,* and *KKK*) stimulated SRE-luciferase in a manner comparable to the myc-Gα_12_^QL^ positive control (Figure 4A). With the exception of mutant *VV*, immunoblot analysis of HEK293 cell lysates revealed expression levels of these mutants similar to myc-Gα_12_^QL^ (Figure 4B).

**Table 2:** **Gα**_**12 **_**cassette mutants corresponding to rgRGS contact points within Gα**_**13**_

**Gα**_**13 **_**residues in contact with p115-RH**	**myc-Gα**_**12**_^**QL **^**NAAIRS mutant**
Val^98^	Q
Asp^101^, Ala^102^	R
Lys^105^, Leu^106^	S
Thr^127^, Arg^128^	V
Phe^168^	BB
Arg^200^, Pro^202^, Lys^204^	HH
Gln^226^	LL
Arg^230^, Lys^231^, Phe^234^	MM
Met^257^	QQ
Arg^260^	RR
Asn^270^	SS
Ile^271^, Glu^273^, Thr^274^, Ile^275^	TT
Asn^278^, Arg^279^, Val^280^	UU
Arg^335^	DDD

Gα_13_-native residues previously identified as providing contact points with the RH domain of p115RhoGEF [20,21] are indicated in the left column. Cassette mutants (“NAAIRS” substitution) in which the homologous residue(s) within Gα_12_ have been altered are indicated in the right column. See Figure 2 for Gα_12_ mutant designations.

### Conformational activation of RhoGEF-uncoupled Gα_12_ mutants

**Figure 5:**
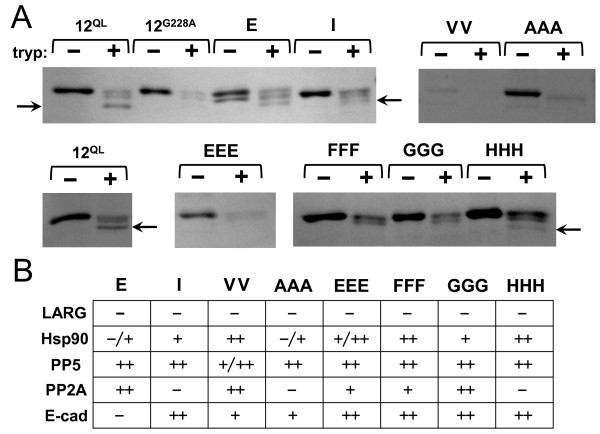
**Conformational efficacy of N-terminal and C-terminal Gα**_**12 **_**mutants uncoupled from RhoGEFs.** (**A**) Trypsin protection of selected Gα_12_ mutants. HEK293 cell lysates expressing the indicated variants of myc-Gα_12_^QL^, or unmodified myc-Gα_12_^QL^ (*12*^*QL*^), or the G^228^A variant of myc-Gα_12_ (12^*G228A*^) were subjected to trypsin protection assays as described in Methods. Samples were incubated 20 min at 30°C in the presence (**+**) or absence (**−**) of TPCK-treated trypsin, and were analyzed by SDS-PAGE and immunoblotting using J169 antibody (1:700 dilution). Small horizontal arrows indicate position of the trypsin-protected fragment in selected lanes. Data presented are representative of two or more independent experiments per sample. (**B**) Specificity of uncoupling in selected Gα_12_ variants. For each cassette mutant of myc-Gα_12_^QL^ (indicated at top), interaction with each Gα_12_ target (indicated at left) was quantified as a pulldown:load ratio as described in Methods, and was calculated as a percent of the identical ratio determined for myc-Gα_12_^QL^ within the same experiment. Values are indicated as follows: (**++**) = >60%, (**+**) = 20 to 60%, (−) = 0 to 20%. Interacting proteins are GST fusions of the following: RH domain of LARG (*LARG*), C-terminal 107 amino acids of heat shock protein-90 alpha (*Hsp90*), protein phosphatase-5 (*PP5*), scaffolding Aα subunit of protein phosphatase-2A (*PP2A*), C-terminal 98 amino acids of E-cadherin (*E-cad*). Values presented indicate the mean of two or more trials per interaction sample.

A concern in our experimental approach was that specific “NAAIRS” cassette substitutions could cause global disruption of Gα_12_ shape, so that a mutant might fail to assume an activated conformation. For RhoGEF-uncoupled Gα_12_ mutants at the N-terminus (i.e. upstream of the P-loop) and C-terminus, we measured protection against trypsin proteolysis. Exchange of GDP for the activating GTP on Gα proteins triggers a conformational change that conceals a trypsin cleavage site within the Switch II region; this property allows the activated state of the Gα protein to be revealed by resistance to trypsin [25,32]. As shown in Figure 5A, mutants *E*, *I*, and *HHH* yielded a protected fragment of approximately 40 kDa that matched the fragment observed following tryptic digestion of myc-Gα_12_^QL^. Results for mutant *AAA* were difficult to interpret; a band of slightly smaller size than undigested *AAA* was generated by tryptic digestion, but it was unclear whether this matched the ~40 kDa trypsin-protected fragment in myc-Gα_12_^QL^. Other C-terminal mutants we tested− *VV, EEE*, *FFF*, and *GGG*− appeared to match the constitutively inactive myc-Gα_12_^G228A^ which lacked this ~40 kDa fragment (Figure 5A). These results suggest several C-terminal mutants of Gα_12_ were sufficiently distorted in shape by the “NAAIRS” substitution to allow trypsin access to proteolytic sites normally not exposed in the GTP-bound state. However, cassette mutants *E* and *I* at the N-terminus and *HHH* at the C-terminus appeared to maintain an activated conformation despite their impairment in RhoGEF binding and SRE stimulation.

We also tested whether RhoGEF-uncoupled cassette mutants at the N- and C-termini of Gα_12_ could interact *in vitro* with other reported binding partners: heat shock protein-90, protein phosphatase-5, the scaffolding Aα subunit of protein phosphatase-2A, and the cytoplasmic tail of E-cadherin [33-36]. As shown in Figure 5B, each mutant displayed pulldown:load ratios >60% of the positive control, myc-Gα_12_^QL^, for at least two of these non-RhoGEF targets. Taken as a whole, these findings reveal a subset of mutations at the N- and C-terminus that selectively uncouple Gα_12_ from RhoGEFs while preserving conformational activation and ability to bind other downstream proteins.

**Figure 6:**
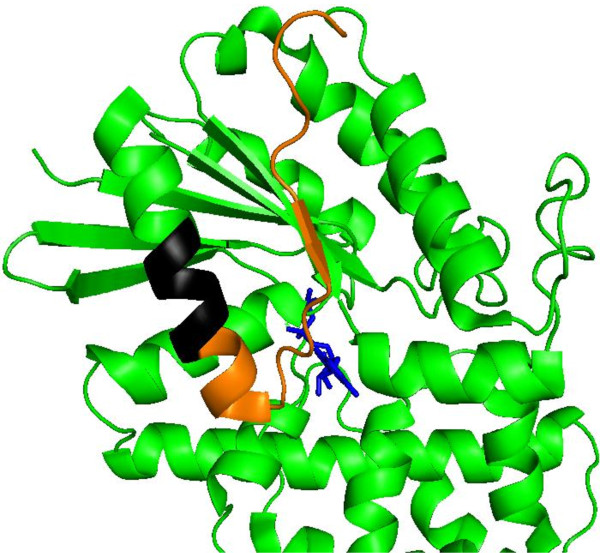
**Structural position of Gα**_**12 **_**C-terminal determinants of RhoGEF binding.** The structure of N-terminally Gαi_1_-substituted Gα_12_ (PDB accession code 1ZCA, [22]) as a GDP•AlF_4_^**¯**^ activated complex was analyzed using PyMOL software. The native Gα_12_ region substituted for the sequence “NAAIRS” in the C-terminal mutants *EEE*, *FFF*, and *GGG* is highlighted in orange, and the sextet substituted in mutant *HHH* is highlighted in black. The bound GDP molecule is highlighted in blue. Figure was rendered in The PyMOL Molecular Graphics System, Version 1.5.0.1 Schrödinger, LLC.

We next visualized the position of these RhoGEF-interacting regions in the crystal structure of a Gα_12_ chimera in which the N-terminal 48 residues were replaced by the N-terminus of Gαi_1_[22]. The native region of Gα_12_ replaced in cassette mutant *E* is not ordered in this structure; however, the regions replaced in the C-terminal mutants *EEE-HHH* are highlighted (Figure 6). The sextet replaced in mutant *HHH* (highlighted in black) resides in the α_5_ helix that extends along the Gα_12_ surface and approaches the C-terminus at the top of the diagram.

### Differential uncoupling of Gα_12_ from LARG and p115RhoGEF

**Figure 7:**
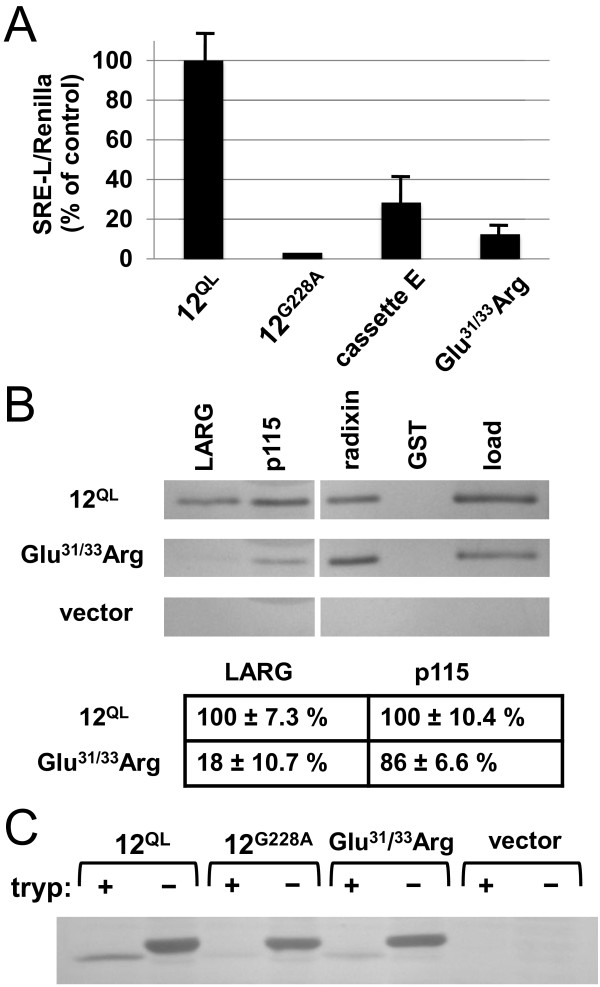
**Selective RhoGEF uncoupling by N-terminal charge substitutions in Gα**_**12**_**. **(**A**) Luciferase reporter gene assays. Cassette mutant *E* (see Figure 2) and the double charge substitution mutant Glu^31/33^Arg were compared to myc-Gα_12_^QL^ (*12*^*QL*^) in SRE-luciferase assays under the cell transfection conditions described in Figure 4. A constitutively GDP-bound variant of wildtype myc-Gα_12_ (*12*^*G228A*^) was assayed in parallel as a negative control. Results shown are the mean of three independent experiments, and error bars indicate range. (**B**) Protein-protein interaction assays. Detergent-soluble extracts from transfected HEK293 cells transfected with myc-Gα_12_^QL^, the Glu^31/33^Arg mutant, or empty pcDNA3.1 plasmid (*vector*) were subjected to co-precipitation assays as described in Methods, using GST-fusions of either LARG-RH (*LARG*), p115RhoGEF-RH (*p115*), the N-terminal domain of the Gα_12_ target radixin [46], or no adduct (*GST*). Prior to the precipitation step, 5% of each lysate was set aside as starting material (*load*). Table values show the pulldown:load ratio for Glu^31/33^Arg as a percent of the positive control value (*12*^*QL*^), with mean +/- range presented for three independent experiments. (**C**) Trypsin protection of the Glu^31/33^Arg mutant, in comparison to constitutively GTP- and GDP-bound Gα_12_. Assays were performed as described in Methods. Results shown are representative of two independent experiments.

We next sought to identify specific residues within these N- and C-terminal sextets of Gα_12_ that mediate RhoGEF interaction. To examine putative surface residues, we performed charge substitutions in the native regions corresponding to cassette mutants *E*, *I*, and *HHH*, and examined these variants for SRE-luciferase activation. None of the single-residue charge-reversals in the regions encompassed in mutants *I* or *HHH* caused significant decrease in SRE signaling (data not shown). However, a double charge-reversal in the mutant *E* region, converting Glu^31^ and Glu^33^ to Arg residues, caused a near-complete loss of SRE activation in HEK293 cells despite normal levels of protein expression (Figure 7A). We next examined this Gα_12_ mutant, designated Glu^31/33^Arg, for binding to the RH domains of LARG and p115RhoGEF. As shown in Figure 7B, a selective loss of RhoGEF binding was observed: the Glu^31/33^Arg charge-reversals severely disrupted LARG-RH binding relative to non-mutated myc-Gα_12_^QL^ (pulldown:load ratio ~18% of control) but had minimal effect on p115-RH binding (ratio ~86% of control). In trypsin protection assays, the Glu^31/33^Arg mutant yielded a protected fragment at the same molecular weight (~40 kDa) as observed for the myc-Gα_12_^QL^ positive control, suggesting its ability to attain an activated conformation (Figure 7C). The intermediate intensity of this band (approximately a midpoint between activated Gα_12_ and the constitutively inactive Gly^228^Ala variant) may be due in part to the mutational introduction of Arg residues providing additional sites for trypsin proteolysis. Taken as a whole, these findings not only provide evidence that the structurally uncharacterized N-terminus of Gα_12_ plays a role in its functional interaction with RhoGEFs, but also reveal individual charged residues in this region as candidates for conferring specificity of Gα_12_ for LARG among the RH-containing RhoGEFs.

## Discussion

The G12 subfamily members Gα_12_ and Gα_13_ are well-documented as utilizing RhoGEFs as downstream signaling effectors. Crystallographic studies by Chen et al. [20] and Hajicek et al. [21] have provided intricate structural details of the interaction between Gα_13_ and the RH domain of p115RhoGEF, identifying a set of Gα_13_ residues that directly contact this target protein. The structure of Gα_12_ also has been elucidated, using a chimera comprised of amino acids 49–379 of Gα_12_ preceded by amino acids 1–28 of Gαi_1_[22]. However, a Gα_12_:RhoGEF complex has not been reported. In the current study, we utilized *in vitro* and cell-based approaches to examine the interaction between Gα_12_ and two putative target RhoGEFs, LARG and p115RhoGEF. Using immobilized RGS-homology (RH) domains of these RhoGEFs, we identified several substitutions of native amino acids in Gα_12_ that disrupted its binding to these proteins and blocked its ability to stimulate the Rho-dependent process of SRE-mediated transcription. Although our results indicated that a number of common determinants in Gα_12_ and Gα_13_ mediate RhoGEF binding, several RhoGEF-uncoupling mutations in Gα_12_ did not correspond to regions of RhoGEF contact within Gα_13_; these include amino acid sextet substitutions in the C-terminal α_5_ helix as well as the structurally uncharacterized N-terminus. Several of these Gα_12_ mutants exhibited protection from tryptic digestion as well as unimpeded binding to other, non-RhoGEF targets, indicating their impaired interaction with RhoGEFs is not caused by failure to attain an activated conformation and suggesting the shapes of other effector-binding surfaces in these Gα_12_ mutants remain intact as RhoGEF interaction is disrupted.

Although Gα_12_ and Gα_13_ share 67% amino acid identity and bind several common downstream targets, several functional differences between these Gα proteins suggest their signaling mechanisms are not redundant [1,3]. Both Gα_12_ and Gα_13_ bind LARG and p115RhoGEF [10,12], and both of these RhoGEFs accelerate GTPase activity of purified Gα_12_ and Gα_13_ in single-turnover assays [13,17]. Whereas Gα_13_ stimulates both p115RhoGEF and LARG to trigger guanine nucleotide exchange on RhoA *in vitro*, Gα_12_ can only stimulate LARG under these experimental conditions, and in a manner dependent on prior phosphorylation of LARG by the tyrosine kinase Tec [10,13]. Also, activated Gα_12_ is more potent than Gα_13_ in recruiting the RH domain of p115RhoGEF to the plasma membrane, and specific mutations in p115RhoGEF disrupt Gα_12_ but not Gα_13_ in triggering this localization [37]. At the cellular and organismal levels, it is increasingly clear that Gα_12_ and Gα_13_ utilize non-overlapping signaling pathways. Mice lacking Gα_13_ die early in embryogenesis due to defects in vascular development and thrombin-induced cell migration, but mice lacking Gα_12_ do not display these developmental defects. However, knockout of Gα_12_ combined with absence of Gα_13_ causes earlier lethality than Gα_13_ knockout alone, and in mice lacking one Gα_13_ allele, at least one Gα_12_ allele must be present for normal embryonic development [38,39]. Furthermore, LPA-induced activation of mTOR complex 2 leading to activation of PKC-δ requires Gα_12_ but not Gα_13_[40]. Because of these differences, plus the increasing list of Gα_12_-specific effector proteins (including another RhoGEF, AKAP-Lbc, that is activated exclusively by Gα_12_ within the G12/13 subfamily), we believe the Gα_12_:RhoGEF interface cannot be defined summarily by structural features of the Gα_13_:RhoGEF complex.

Among the Gα_13_ residues that provide contact points with p115RhoGEF in crystallographic studies [20,21], many have corresponding residues within Gα_12_, and therefore we paid particular attention to Gα_12_ cassette mutants corresponding to these key Gα_13_ residues (see Table 2). For example, the Gα_12_ mutant *HH* replaced residues corresponding to Gα_13_ residues Arg^200^, and Lys^204^, both of which provide contact points with p115-RH. In another Gα_12_ cassette mutant, termed *RR,* a substituted residue corresponds to Arg^260^ within Gα_13_; this residue provides a key contact with amino acids within the βN-αN region of p115RhoGEF. Also, Gα_12_ cassette mutants *Q, R,* and *S* contain altered residues in the Gα_12_ helical domain that correspond to p115-RH interacting residues in Gα_13_. Among the Gα_12_ mutants corresponding to p115-RH contact points in Gα_13_, most showed impaired RhoGEF interaction and poor stimulation of SRE-mediated signaling. However, several differences between Gα_12_ and Gα_13_ were noted, particularly in the helical domain. Gα_12_ cassette mutant *V* alters residues that correspond to two contact points within the Gα_13_:p115-RH complex; however, this mutant showed minimal impairment in RhoGEF binding *in vitro* and stimulated SRE-mediated transcription robustly in cells. Gα_12_ mutant *BB*, which removes a Phe corresponding to a Gα_13_ contact point with p115-RH, displayed a slight impairment in SRE-mediated transcriptional activation and no impairment of RhoGEF binding. In addition, Gα_13_ utilizes a C-terminal residue (Arg^335^) as a contact point with p115-RH, but the corresponding Gα_12_ cassette mutant (*DDD*) exhibited normal binding to RhoGEFs and only modest impairment in SRE signaling. However, because this cassette mutant preserves the corresponding Arg residue in Gα_12_ (DRKRRN substituted for NAAIRS), it is possible this Arg in Gα_12_ participates in RhoGEF binding despite the alteration in adjacent amino acids.

Aside from the N- and C-terminal mutants of Gα_12_ that show impaired RhoGEF binding, we have identified other RhoGEF-uncoupling mutations in Gα_12_ that lack corresponding Gα_13_ contact points for p115-RH (see Tables 1 and 2). None of the native Gα_12_ residues replaced in cassette mutants *M* and *Z* match p115-RH contact points in Gα_13_, and thus may indicate Gα_12_-specific determinants of RhoGEF interaction. Impaired RhoGEF binding also was observed in Gα_12_ mutants *J* and *K*; however, this most likely was due to these substitutions disrupting the canonical GXGXXGKS guanine nucleotide binding motif [41]. Although our results suggest a core similarity in the mechanisms utilized by Gα_12_ and Gα_13_ to engage RhoGEF targets, it is apparent that several determinants of RhoGEF binding are unique to Gα_13_. We have identified determinants that may be unique to Gα_12_ or potentially important for both G12/13 subfamily members in RhoGEF engagement. Studies of Gα_13_ variants harboring corresponding mutations will be important in distinguishing these possibilities.

A role for the C-terminus of G12/13 subfamily proteins in RhoGEF engagement has been suggested by prior studies. Kreutz et al. [42] engineered chimeras of Gα_12_ and Gα_13_ that were interchanged downstream of the Switch III region, and demonstrated the C-terminal 114 amino acids of Gα_13_ as sufficient for its unique ability to stimulate purified p115RhoGEF to activate RhoA. Also, a chimeric Gα_13_ in which the region downstream of Switch III was replaced by the corresponding region of Gαi_2_ displayed loss of ability to stimulate SRE-mediated transcriptional activation [43]. Initial crystallographic studies of Gα_13_:RhoGEF interaction utilized a chimeric Gα_13_ harboring Gαi_1_ sequence at the C-terminus, and determinants of RhoGEF binding were not found downstream of the Switch regions in this protein [20]. Subsequent crystallographic work utilizing Gα_13_ with native C-terminal sequence did identify residues slightly downstream of the Switch III region as critical for RhoGEF engagement [21], and also revealed a more distal residue in the C-terminal region (Arg^335^) as providing a contact point with the RH domain of p115RhoGEF. However, no residues at the extreme C-terminus of Gα_13_, including the α_5_ helix, were found to mediate RhoGEF binding. Our results suggest differences between Gα_12_ and Gα_13_ in the role of the C-terminus, as several substitutions near the extreme C-terminus of Gα_12_ disrupted RhoGEF interaction, most notably the cassette mutant *HHH* within the α_5_ helix.

The N-terminus provides the greatest amino acid sequence divergence between Gα_12_ and Gα_13_. Gα subunits utilize this region for interaction with Gβγ [44], and in Gα_12_ and Gα_13_ this region confers specificity of coupling to thrombin and LPA receptors, respectively [19]. Importantly, Gα_13_ is a more potent stimulator of RhoGEF activation *in vitro* than a chimeric Gα_13_ harboring the N-terminus of Gαi_1_, indicating a possible role of the Gα_13_ N-terminus in RhoGEF activation [21]. However, specific determinants within the N-terminus of G12/13 subfamily proteins that mediate binding to effectors, including RhoGEFs, have not been reported. The 48-residue region at the N-terminus of Gα_12_ has not been characterized in crystallographic studies, because its replacement by the Gαi_1_ N-terminus was necessary for obtaining sufficient quantities of purified protein [16,22]. Furthermore, the N-terminus was disordered in crystallographic analysis of both the aforementioned Gαi_1_/Gα_13_ hybrid and a more recent structure of full-length Gα_13_[21], suggesting the Gα_12_ N-terminus may be refractory to crystallographic analysis even if native sequence is utilized. Our approach of employing cassette substitution mutants throughout the length of Gα_12_ has provided an indirect means of circumventing this obstacle, and has revealed specific N-terminal regions as possible determinants of RhoGEF interaction. Importantly, our discovery that mutations in this N-terminal region (cassette mutants *E* and *I*) cause loss of RhoGEF binding allowed us to focus on putative surface residues in these substituted regions, ultimately revealing Glu^31^ and Glu^33^ as critical for Gα_12_ interaction with LARG and stimulation of SRE-mediated transcription. Our finding that charge substitutions of these N-terminal Gα_12_ residues disrupted binding to the LARG-RH domain but had minimal effect on interaction with the corresponding domain of p115RhoGEF was intriguing, and suggested these residues play a role in targeting Gα_12_ preferentially to LARG. It is possible that Gα_12_ harbors sufficient RhoGEF-interacting surfaces for *in vitro* binding to p115RhoGEF, but that a functional, physiological interaction (i.e. with LARG) requires this N-terminal region. Our RhoGEF binding results for Gα_12_ cassette mutant *E*, as well as the more specific Glu^31/33^Arg mutant, were surprising in light of earlier findings that RhoGEF binding was preserved in a Gα_12_ chimera containing the Gαi_1_ N-terminus [22]. It is possible that “NAAIRS” substitution and particularly the Glu^31/33^Arg charge-reversals cause a more dramatic change to this RhoGEF binding surface than occurs when Gαi_1_ sequence is introduced. Cassette mutant *E* and the Glu^31/33^Arg mutant are impaired in activating the Rho-dependent readout of SRE-mediated transcriptional activation in cells, and it remains to be determined whether the Gαi_1_/Gα_12_ chimera is similarly impaired in stimulating this pathway.

Because previous phosphorylation of LARG by Tec is a requirement for Gα_12_, but not Gα_13_, for *in vitro* activation of Rho, it will be important to determine whether this phosphorylation event regulates interaction of LARG with Gα_12_, particularly its N-terminus and C-terminal α_5_ helix. Furthermore, as suggested by Hajicek et al. [21], it is conceivable that post-translational modification of p115RhoGEF in cells modulates its responsiveness to Gα_13_ or could potentially render it a target of Gα_12_. A challenge for future studies of Gα_12_- and Gα_13_-mediated signaling will be to determine the combinations of G12/13 subfamily α-subunits and RhoGEFs that activate Rho in response to different signaling inputs, and in different cell and tissue types.

## Conclusions

Gα_12_ and Gα_13_ define the G12/13 class of heterotrimeric G protein α-subunits, which participate in numerous signaling pathways through stimulation of RhoGEFs that subsequently activate Rho. Although these proteins are non-redundant in their stimulation of effectors and their cellular and organismal roles, only Gα_13_ has been characterized in the structural basis of its interaction with RhoGEF targets. However, the involvement of Gα_12_ in stimulating SRE-mediated transcription, cell rounding, c-Jun N-terminal kinase activation, cell growth, and metastatic invasion supports a physiological role for a Gα_12_-RhoGEF-Rho axis in developmental pathways and disease progression [45]. Therefore, an improved understanding of the structural aspects of Gα_12_:RhoGEF interaction likely will be of broad importance. Our results provide several key additions to this structural model: 1) characterization of the Gα_12_:RhoGEF interacting surface by identifying regions in Gα_12_ that mediate binding; 2) unexpected roles of the Gα_12_ N-terminal region and C-terminal α_5_ helix in engagement of RhoGEFs; 3) identification of specific residues near the Gα_12_ N-terminus that may mediate its selectivity for LARG as an effector protein. To date, no structural studies have examined the interaction of Gα_12_ with RhoGEFs. Our hope is that mutant-based strategies will augment such crystallographic approaches and provide key details toward understanding the structural aspects and biological role of this Gα:effector interaction.

## Methods

### DNA constructs

Plasmids encoding 1) a fusion of glutathione-S-transferase (GST) to amino acids 320–606 of LARG (GST-LARG-RH), and 2) amino acids 1–252 of p115RhoGEF with an N-terminal myc epitope tag were kindly provided by Tohru Kozasa (Univ. of Ill., Chicago). We used PCR to subclone the p115RhoGEF sequence into pGEX-2T (GE Healthcare) to produce GST-p115-RH. All “NAAIRS” amino acid substitution mutants within myc-tagged Gα_12_ Gln^229^Leu (myc-Gα_12_^QL^) were engineered as described previously [29]. Single amino acid substitutions were engineered in myc-Gα_12_^QL^ using the QuikChange II® site-directed mutagenesis system (Agilent Technologies), and this system was used to engineer a constitutively inactive Gly^228^Ala variant (myc-Gα_12_^G228A^) within a plasmid encoding myc-tagged, wildtype Gα_12_ (provided by Pat Casey, Duke University). The luciferase reporter plasmid SRE-L was a gift from Channing Der (University of North Carolina Chapel Hill).

### Expression and immobilization of GST fusion proteins

GST fusion constructs were transformed into BL21(Gold)-DE3 cells (Stratagene). Cells were grown under 75 μg/ml ampicillin selection to OD_600_ of 0.5−0.7, and recombinant protein expression was induced using 0.5 mM isopropyl-β-D-thiogalactopyranoside (Fisher Scientific). After 3 h, cells were lysed on ice using 0.32 mg/ml lysozyme (MP Biomedicals), and GST fusion proteins were bound to glutathione-sepharose 4B (GE Healthcare) as described previously [31,34]. Following three washes in 50 mM Tris pH 7.7 supplemented with 1 mM EDTA, 1 mM dithiothreitol, and 150 mM NaCl, samples were snap-frozen in aliquots and stored at −80°C.

### Preparation of detergent-soluble extracts harboring Gα_12_ mutants

Human embryonic kidney cells (HEK293) were grown in Dulbecco’s modified Eagle medium (Mediatech, Manassas, VA) supplemented with 10% fetal bovine serum (Hyclone, Logan, UT), penicillin and streptomycin. For myc-Gα_12_^QL^ and each of its 62 NAAIRS substitution mutants (see Figure 2), 7.0 μg of plasmid DNA was transfected into a 10-cm dish of HEK293 cells grown to approximate 90% confluence, using Lipofectamine 2000 (Invitrogen) according to the manufacturer’s instructions. After 36–42 hours, cells were scraped from dishes, washed twice with phosphate-buffered saline, and solubilized in NAAIRS Lysis buffer (50 mM HEPES pH 7.5, 1 mM EDTA, 3 mM dithiothreitol, 10 mM MgSO_4_, 1% (w/v) polyoxyethylene-10-lauryl ether) containing the protease inhibitors 4-(2-aminoethyl)benzenesulfonyl fluoride hydrochloride (1.67 mM), leupeptin (2.1 μM), pepstatin (1.45 μM), TLCK (58 μM), TPCK (61 μM), and phenylmethylsulfonyl fluoride (267 μM). Samples were centrifuged at 80,000 *g* for 1 h, and supernatants were snap-frozen in 60-μl aliquots and stored at −80°C.

### Protein interaction assays

HEK293 cell extracts were diluted in NAAIRS Lysis buffer lacking polyoxyethylene-10-lauryl ether, using sufficient volume to dilute this detergent in the samples to 0.05% (w/v). Next, sepharose-bound GST fusion proteins were added and allowed to incubate for approximately 2 h at 4°C with continuous inversion. A percentage of the diluted extract was set aside as starting material prior to sepharose addition. Next, samples were centrifuged at 1,300 *g*, and pellets were washed three times and then subjected to SDS-PAGE and immunoblot analysis using an antibody specific to the Gα_12_ N-terminus (Santa Cruz Biotechnology) or the myc 9E10 epitope tag (Zymed), followed by alkaline phosphatase conjugated secondary antibodies (Promega). For each variant of myc-Gα_12_^QL^, the Gaussian intensity of the ~45 kDa band from the precipitated material and the corresponding band from the starting material were quantified using a Kodak Gel Logic 100 system equipped with Molecular Imaging 5.X software (Carestream Health, New Haven CT).

### Reporter gene assays

HEK293 cells grown in 12-well plates were transfected with 0.2 μg SRE-luciferase plasmid (encoding firefly luciferase) and 0.02 μg pRL-TK plasmid encoding *Renilla* luciferase, plus plasmids encoding variants of myc-Gα_12_^QL^. Reporter assays for SRE-mediated transcriptional activation were performed as described previously [31]. Briefly, cells were washed with phosphate-buffered saline and lysed in 1X passive lysis buffer (Promega), and lysates were analyzed using a Dual-luciferase assay system and GloMax 20/20 luminometer (Promega). Light output due to firefly luciferase activity was divided by output from *Renilla* luciferase activity to normalize samples for transfection efficiency.

### Trypsin protection experiments

HEK293 cells grown in 10-cm dishes were transfected with various Gα_12_ constructs using Lipofectamine 2000 (Invitrogen), and tryptic digestions were performed as a modification of the procedure of Kozasa and Gilman [25]. Briefly, cells were lysed in 50 mM Hepes pH 8.0, 1 mM EDTA, 3 mM dithiothreitol, 1% polyoxyethylene-10-lauryl ether containing the same protease inhibitors as NAAIRS Lysis buffer (see above) but at two-fold lower concentration. Samples were cleared by centrifugation at 70,000 *g* for 1 h, and supernatants were diluted 20-fold in volume using 50 mM Hepes pH 8.0, 1 mM EDTA, 3 mM dithiothreitol, 10 mM MgSO_4_. Samples were digested with 10 μg/ml TPCK-treated trypsin (New England Biolabs) for 20 min at 30°C, and proteolysis was terminated by addition of 100 μg/ml lima bean trypsin inhibitor (Worthington, Lakewood NJ). Samples were analyzed by SDS-PAGE and immunoblotting using J169 antisera specific to the Gα_12_ C-terminus, provided by Tohru Kozasa (Univ. of Ill., Chicago).

## Abbreviations

Gα12 and Gα13: Heterotrimeric guanine nucleotide binding protein α-subunits of the G12/13 subfamily; GST: Glutathione-S-transferase; HEK: Human embryonic kidney; LARG: Leukemia-associated RhoGEF; LPA: Lysophosphatidic acid; NAAIRS mutant: Variant of Gα_12_ in which a consecutive sextet of native residues has been replaced by Asn-Ala-Ala-Ile-Arg-Ser; RGS: Regulator of G protein signaling; RH: RGS homology; RhoGEF: Rho-specific guanine nucleotide exchange factor; SRE: Serum response element.

## Competing interests

The authors declare that they have no competing interests.

## Authors’ contributions

BJR and WCS participated in design of the study, carried out PCR-based mutagenesis, designed and executed protein interaction screens, and participated in drafting the manuscript. In addition, BJR performed reporter assays and WCS performed 3-D protein imaging and analysis. ERM, ESF, TYC, and CMO engineered various GST-fusion constructs and carried out protein interaction screens. LAF participated in initial design of the study and carried out pilot experiments. TEM conceived of the study, participated in its design, coordination, engineering of constructs and data collection, and drafted the manuscript. All authors have read and approved the final manuscript.

## Authors’ information

TEM is an affiliate member of the UNC Lineberger Comprehensive Cancer Center (Chapel Hill, NC).
